# TRIC-A regulates intracellular Ca^2+^ homeostasis in cardiomyocytes

**DOI:** 10.1007/s00424-021-02513-6

**Published:** 2021-01-21

**Authors:** Xinyu Zhou, Ang Li, Pei-hui Lin, Jingsong Zhou, Jianjie Ma

**Affiliations:** 1 Department of Surgery, The Ohio State University Columbus, Columbus, OH 43210 USA; 2 Department of Kinesiology, College of Nursing and Health Innovation, University of Texas at Arlington, Arlington, 76019 USA

**Keywords:** Heart, RyR, Mitochondria, Nuclear envelope

## Abstract

Trimeric intracellular cation (TRIC) channels have been identified as monovalent cation channels that are located in the ER/SR membrane. Two isoforms discovered in mammals are TRIC-A (TMEM38a) and TRIC-B (TMEM38b). TRIC-B ubiquitously expresses in all tissues, and TRIC-B^−/−^ mice is lethal at the neonatal stage. TRIC-A mainly expresses in excitable cells. TRIC-A^−/−^ mice survive normally but show abnormal SR Ca^2+^ handling in both skeletal and cardiac muscle cells. Importantly, TRIC-A mutations have been identified in human patients with stress-induced arrhythmia. In the past decade, important discoveries have been made to understand the structure and function of TRIC channels, especially its role in regulating intracellular Ca^2+^ homeostasis. In this review article, we focus on the potential roles of TRIC-A in regulating cardiac function, particularly its effects on intracellular Ca^2+^ signaling of cardiomyocytes and discuss the current knowledge gaps.

## Introduction

Ca^2+^ signaling plays a central role in cardiac physiology [18]. The regulation of Ca^2+^ signaling shows a great dynamic range in terms of frequency and spatial-temporal relationship, forming a variety of patterns from localized brief Ca^2+^ bursts to long-lasting, global Ca^2+^ transients [8, 17, 18, 53, 68, 81]. On the other hand, sustained elevation of intracellular Ca^2+^ is known to be cytotoxic, which would lead to mitochondria damage, dysregulation of gene expression, and apoptotic and necrotic cell death. Thus, physiological Ca^2+^ homeostasis and signaling must be tightly regulated. To assure this, many molecules and proteins, including signaling ligands and receptors, are coupled together and function in a coordinated manner [6, 7]. Compromise of such Ca^2+^ homeostasis and signaling has been linked to different human diseases, including muscle dysfunction and heart failure [8, 17, 53, 68, 81].

In cardiomyocytes, rhythmic electrical excitation travelling through the cellular membrane triggers the entry of Ca^2+^ through L-type Ca^2+^ channels that mediates the systolic release of Ca^2+^ from the sarcoplasmic reticulum (SR) to the cytosol through ryanodine receptor 2 (RyR_2_) channels [7, 12, 18, 95]. One of the fundamental goals of cardiac physiology is to understand the detailed mechanisms behind Ca^2+^ handling and to find approaches to correct defective Ca^2+^ cycling associated with heart failure and arrhythmias.

Trimeric intracellular cation (TRIC) channels have been identified as monovalent cation channels that are located in the ER/SR membrane [101, 109]. Two isoforms discovered in mammals are TRIC-A (TMEM38a) and TRIC-B (TMEM38b). While TRIC-B ubiquitously expresses in all tissues, TRIC-A mainly expresses in excitable cells. The aggravated embryonic lethality in TRIC-A and TRIC-B double knockout animals suggests the crucial role of TRIC channels in embryonic development [101]. TRIC-A^−/−^ mice survive normally but show abnormal SR Ca^2+^ handling in skeletal muscle, characterized by irregular contractile force during fatigue, compromised Ca^2+^ sparks, and Ca^2+^ overload in the SR [105]. Additionally, TRIC-A ablation causes hypertension in mice due to altered Ca^2+^ signaling in smooth muscle [98]. On the other hand, the absence of TRIC-B is lethal in mice at the neonatal stage [101]. Later studies demonstrated the crucial function of TRIC-B in inositol trisphosphate receptor (IP_3_R)–mediated Ca^2+^ signaling from the endoplasmic reticulum (ER) in non-excitable cells [97]. Takeshima’s group demonstrated the important function of TRIC-B in the development of bone and identified several genetic mutations within the TRIC-B locus that are associated with osteogenesis imperfecta [40, 107].

The crystal structure of TRIC has been resolved and provided insights into their functional mechanism [43, 65, 82, 91, 100]. Yang et al. resolved the nematode TRIC homologs and demonstrated that the channel consists of seven transmembrane domains, predicted by a previous bioinformatic approach [79], and forms a homotrimeric complex within the lipid membrane [100]. Each monomer contains an hourglass-shaped, fluid-filled, cation-permeable pore. This important feature has subsequently been confirmed in the structures of other types of prokaryotic TRIC channels [43, 82], and in vertebrate TRIC-A and TRIC-B channels [91].

## TRIC-A functions as a counter-current channel in SR/ER Ca^2+^ signaling

During excitation-contraction (EC) coupling, the opening of RyR allows a massive amount of Ca^2+^ moving from the SR to the cytosol due to the electrochemical gradient of Ca^2+^ ions. Since Ca^2+^ is a cation, this instant efflux of Ca^2+^ would result in the accumulation of negative charges inside the SR. This potential asymmetry would hinder the subsequent Ca^2+^ release. Similarly, during relaxation, the uptake of Ca^2+^ into the ER/SR also requires a similar opposite ionic counter movement. In the absence of an additional flow of counter-ions to balance the charge, the Ca^2+^ release or uptake during EC coupling would be compromised. Thus, robust counter-ion flux across the SR/ER is crucial to compensate the potential changes and promotes efficient Ca^2+^ release and uptake during EC coupling [9, 19, 27, 28, 85, 89, 90]. Identification of the molecular identity of those counter-ion channels are crucial for understanding the Ca^2+^ regulation and may provide potential molecular targets for developing therapeutic means for cardiac and skeletal muscle diseases.

Yazawa et al. first unveiled the molecular identity of such K^+^-permeable counter-ion channels as trimeric intracellular cation channels (TRICs) [101]. Sitsapesan’s group conducted several studies concerning the biophysical characteristics of TRIC-A and TRIC-B channels. They found that TRIC-A displayed different conductance properties [67, 88]. Unlike TRIC-A, TRIC-B preferably opened at sub-conductance levels [88]. Further experiments unveiled other differences between these two isoforms. For TRIC-A channels, the conductance property did not change regardless of whether they were isolated or clustered in space. But for TRIC-B channels, grouped trimmers displayed a much higher open probability comparing to isolated trimmers. Thus, they proposed that physical interactions between multiple TRIC-B trimeric channels changed the gating behavior of the channels [64]. The activation of TRIC-A channel was regulated by both voltage and Ca^2+^ binding [67, 101]. Ca^2+^ regulation of TRIC channels was investigated in depth by Chen’s group in a recent study [91]. At resting state, Ca^2+^ binds to G74 residue on the threefold axis, which is located in the luminal side of TRIC-A, and stabilizes the closure of the pore. During systolic Ca^2+^ release, depletion of luminal Ca^2+^ results in the dissociation of Ca^2+^ binding to the channel. This change enables the Ca^2+^-dependent gating of the TRIC-A channel. They also found K129 was critical for the TRIC-A pore conductance. K129A mutation resulted in constant opening of the channels while TRIC-A channels with K129Q mutation mostly stayed closed. Taken all together, TRIC channels ideally meet all expectations of the counter-current channels for the ER/SR Ca^2+^ release, due to their voltage-dependent, luminal Ca^2+^-regulated, and potassium-permeable characteristics [91].

However, the assumption that TRIC channels carry the essential counter-current for SR charge compensation was challenged by Gillespie and Fill, who argued that RyR channels are poorly Ca^2+^ selective and exhibit high conductance of monovalent and divalent cations such as K^+^ and Mg^2+^, which could carry the counter-current [31]. Using a Poisson-Nernst-Planck/density functional theory (PNP/DFT) model, their calculation demonstrated that large K^+^ and Mg^2+^ counter-current through RyR could clamp the SR membrane potential far from the Nernst potential for Ca^2+^, nullifying the need for additional counter-current channels [30, 31]. Moreover, Guo et al. studied the isolated cardiac SR microsomes and saponin-permeabilized cardiomyocytes. They revealed that replacement of cytosolic K^+^ with Na^+^ or Cs^+^ failed to affect single RyR_2_ channel currents, open probability, or Ca^2+^ sparks. Thus, they rejected the idea that SR K^+^ channels have significant contribution to the counter-current supporting RyR_2_ Ca^2+^ release [35]. On the other hand, recent mathematical modeling suggests that no single channel type is essential for the counter-current. Instead, all possible sources of channel-mediated cation or anion counter-current would be utilized to form a cascading network of counter-currents in the entire SR [112]. This network would ensure an efficient counter-current to support Ca^2+^ release in different physiological conditions. In this case, TRIC-A may not represent an indispensable prerequisite for efficient SR Ca^2+^ release as presumed earlier but still contribute to the ion equilibrium across SR during repetitive cycles of release events. This conclusion, together with the earlier observation that cardiomyocytes from TRIC-A/TRIC-B double knockout mice exhibit significantly elevated caffeine-induced Ca^2+^ release [101], raised an intriguing question: could TRIC channels have additional Ca^2+^ regulatory roles for RyR other than the counter-current function?

## TRIC-A regulates SR Ca^2+^ signaling through direct modulation of RyR activity

RyRs are high conductance Ca^2+^ release channels located on the junctional SR in striated muscles [60]. In addition to small molecules like Ca^2+^, Mg^2+^, and ATP that regulates RyR_2_ directly, many other RyR_2_ modulators have been identified and extensively studied, including FKBP12.6 [83], calmodulin (CaM) [61], sorcin [51], protein kinase A (PKA) [55], protein phosphatase 1 and 2A (PP1, and PP2A) [56], and Ca^2+^/calmodulin-dependent protein kinase type II (CaMKII) [93]. It is worth noting that almost all of those RyR_2_ regulatory proteins function as stabilizers of RyR_2_ activities, whose defects would commonly lead to Ca^2+^ leakage through RyR_2_ channels [4, 14].

A study from Sitsapesan’s group found that in skeletal muscle, the RyR_1_ channel from the TRIC-A^−/−^ mice displayed increased sensitivity to Mg^2+^ inhibition and a defective response to protein kinase A phosphorylation [26]. Meanwhile, physiological activators such as ATP are less effective in activating individual RyR_1_ channels reconstituted into the lipid bilayer membrane. However, they also reported that the Ca^2+^-dependent control of RyR_1_ channel was not altered in the absence of TRIC-A [26]. These findings are consistent with the potential role of TRIC-A as an enhancer of RyR channels, such that the absence of TRIC-A leads to reduced RyR channel function.

Recently, Zhou et al. presented evidence of a direct modulation of RyR_2_ activity by TRIC-A [110]. We observed that cardiomyocytes derived from TRIC-A^−/−^ mice have a lower basal Ca^2+^ spark frequency and a higher SR Ca^2+^ content. These myocytes also have slow-rising and prolonged intracellular Ca^2+^ transient profiles. We further explored the role of TRIC-A in HEK293 cells with inducible expression of RyR_2_ as a model of store-overload induced Ca^2+^ release (SOICR) [41, 110]. Overexpression of TRIC-A in HEK293 cells leads to decreased spontaneous Ca^2+^ oscillations and hence help mitigate SOICR as a result of reduced ER Ca^2+^ content. This phenotype is specific to TRIC-A as co-expression of TRIC-B does not alter the amplitude or kinetics of SOICR in the same setting. Furthermore, our biochemical and immunohistochemical data demonstrated a direct interaction between TRIC-A and RyR_2_, possibly via the carboxyl-terminal tail (CTT) domain of TRIC-A (CTT-A). This result is consistent with the data from Dr. Zorzato’s group where they reported that TRIC-A (named SRP-27 in their paper) directly binds to RyR in their pulled-down and coimmunoprecipitation assays [10]. We further unveiled that CTT-A is a crucial structure responsible for the RyR_2_ modulation as evidenced by both HEK293 SOICR assay as well as measuring the effect of CTT-A on RyR_2_ channel from cardiac SR vesicles in reconstituted lipid bilayer assay [110]. Interestingly, the CTT domains are absent in all of the recent structural studies. The investigators claim this domain may decrease the stability of the crystal [43, 65, 82, 100] and suggest it could be a flexible function domain.

Further computational modeling predicted several potential binding sites of TRIC-A on RyR_2_ [110]. Remarkably, one of the potential binding sites is located at the SPRY domain of RyR. It is known that the SPRY domain of RyR could bind to the dihydropyridine receptor for control of RyR channel activity [21]. Thus, the hypothesized interaction of CTT-A and SPRY domain could be a potential target site for therapeutic regulation of RyR activity. Together, these data revealed a novel role of TRIC-A as a direct modulator of RyR_2_, in addition to its counter-ion channel function. Both functions would enhance RyR_2_-mediated Ca^2+^ release in cardiac muscle.

Mutations in RyR_2_ linked to cardiac arrhythmia, including catecholaminergic polymorphic ventricular tachycardia (CPVT), have been identified as gain of function mutations such as N4104K, R4497C, and N4895D [41, 69]. Those mutations enhance the activity of RyR_2_, elevate Ca^2+^ leakage, and subsequently lead to cardiac dysfunction [4, 41]. Therefore, stabilizing RyR_2_ activity to prevent the leakage of Ca^2+^ has been a major target for the potential treatment of cardiac diseases. Interestingly, several novel CPVT mutations, including I4855M [73] and A4860G [42], have been identified as loss-of-function mutations. Moreover, phosphorylation of RyR_2_ at S2808 has been shown to promote the hyperactivity of RyR_2_ and contribute to the pathological condition of the heart [76]. However, the ablation of the RyR2 phosphorylation at Ser-2808 did not reverse the cardiac phenotypes but rather exacerbated the disease phenotype by reducing the survival rate and impairing in vivo cardiac function [49]. Thus, accumulating evidence shows that RyR_2_ requires tight and balanced regulations not only by negative regulators but also by positive modulators. Either hyper- or de- activation of RyR_2_ could result in pathological consequences. The phenotypes of TRIC-A^−/−^ heart show some similar characteristics to those in RyR_2_-S2808A [87], RyR_2_-A4860G [106], and RyR_2_-Ex3-del^+/−^ mice [50], which further suggests the importance of the proper regulation of RyR_2_ channels. Potential therapeutic interventions can be used to target the functional interaction between TRIC-A and RyR_2_ to restore defective Ca^2+^ signaling in cardiovascular diseases.

The functional crosstalk between IP_3_R and RyR-mediated Ca^2+^ signaling has also been implicated in muscle and heart cells under physiological and pathological conditions [24, 44, 46, 84, 102, 103, 111]. Dissecting the role of TRIC-A and TRIC-B in RyR/IP_3_R crosstalk regulation and Ca^2+^ signaling regulation in physiological and pathological condition will be another essential task of future research.

## TRIC-A regulates SOCE through interaction with STIM1/Orai1 complex

ER/SR Ca^2+^ modulation has been the primary focus of TRIC studies in the past decade. However, a new study from Shrestha et al. unveiled a new role of TRIC-A in the regulation of Ca^2+^ entry mechanism [78]. They found that TRIC-A modulated store operated Ca^2+^ entry (SOCE) by interacting with stromal interaction molecule 1 (STIM1)/Ca^2+^-release activated Ca^2+^ channel 1(Orai1) complex [78]. STIM1 is an ER/SR-resident transmembrane protein with a Ca^2+^ binding domain. Upon [Ca^2+^]_ER_ depletion, dissociation of Ca^2+^ results in STIM1 oligomerization and its translocation to ER–plasma membrane (PM) junctions. These STIM1 clusters would recruit and activate the Orai1 channels on the plasma membrane and trigger Ca^2+^ entry into the cytosol [71, 94]. Using mutant Orai1 or Orai1 blocker BTP2, Shrestha et al. showed that the RyR_2_-induced Ca^2+^ oscillations in HEK293 cells required the SOCE machinery. Upon ER Ca^2+^ store depletion, TRIC-A channels co-clustered with the STIM1/Orai1 complex within ER–PM junctions. The association of STIM1 with TRIC-A reduced the co-localization of STIM1 and Orai in the cells, thus suppressing SOCE. Furthermore, they demonstrated that knocking down of TRIC-A in HL-1 would promote SOCE, mimicking the effects of STIM1 overexpression.

Several studies have established the physiological and pathological relevance of STIM1 in the heart [66, 72]. Silencing of STIM1 results in compromised cardiac function as well as reduced cardiomyocyte size [5]. On the other hand, STIM1 overexpression in the transgenic mice leads to cardiac hypertrophy and sudden death [20]. Although STIM1 is abundantly expressed in neonatal cardiomyocytes as well as in HL-1 cells, the level in adult cardiomyocytes is low [86]. Moreover, Hill’s group demonstrated that SOCE abundantly presents in neonatal cardiomyocytes; however, SOCE is absent in adult cardiomyocytes [52]. Therefore, the role of TRIC-A interaction with STIM1 in the control of SOCE in cardiomyocytes needs further investigation.

## Potential role of TRIC-A in mitochondrial metabolism and SR-mitochondria crosstalk

The crosstalk between intracellular organelles has drawn great attention in cell biology and physiology studies in recent years. The crosstalk between ER/SR and mitochondria-mediated Ca^2+^ signaling plays an important role in physiological and pathological conditions [74, 75]. Li et al. presented evidence implicating TRIC-A in regulation of mitochondrial metabolism through directly or indirectly modulating mitochondrial Ca^2+^ signaling [47]. Mitochondria play a crucial role in oxidative metabolism that produces 95% energy required for the cell function [3]. In cardiomyocytes, Ca^2+^ uptake by mitochondria is an important messenger for matching energy supply to demand during various physiological workloads. This is achieved through Ca^2+^-induced activation of Krebs cycle dehydrogenases and pyruvate dehydrogenase [15, 16, 36, 58]. Additionally, mitochondrial Ca^2+^ has also been indicated to enhance the activities of complexes I, II, IV, and V along the respiratory chain [22, 32]. However, persistent augmentation of Ca^2+^ handling in cardiomyocytes, triggered by repetitive isoproterenol treatment, chronic pressure overload, or ischemia/reperfusion, would lead to pathological mitochondrial Ca^2+^ overload, which is not a simple compensatory mechanism to increase energy output [37, 48, 75, 77, 80, 96, 108]. This pathological overload usually occurs concomitantly with increased production of mitochondrial reactive oxygen species (ROS), which is causally related to progressive heart failure [57, 63]. Moreover, recent studies revealed that mitochondrial Ca^2+^ overload enhanced the activity of Na^+^/Ca^2+^/Li^+^ exchanger (NCLX), leading to Ca^2+^ extrusion at the cost of increased matrix Na^+^. Matrix Na^+^ interacts with phospholipids, such as phosphatidylcholine, of the inner mitochondria membrane, leads to reduced membrane fluidityand, the diffusion of ubiquinol (coenzyme Q) from glyceraldehyde 3-phosphate dehydrogenase (GAPDH) or complex II to complex III of the respiratory chain, and elevation of ROS production of complex III at Qo site [38]. ROS thus activates hypertrophic signaling through the oxidation of histone deacetylase 4 and the activation of other redox-sensitive pathways [1]. Furthermore, ROS impairs EC-coupling by altering the function of RyRs, SR Ca^2+^-ATPase, NCX, and other Ca^2+^ signaling–related proteins [29, 33, 99]. Toxicity of mitochondrial Ca2+ overload is not only about excessive ROS generation but also related to the activation of apoptotic and necrotic cell death. Mitochondrial Ca2+ overload induces sustained opening of the mitochondrial permeability transition pore (mPTP), a pore complex in the IMM that allows passage of ions and solutes up to 1.5 kDa. This sudden increase of IMM permeability causes the loss of mitochondrial membrane potential and promotes the release of pro-apoptotic factors, which triggers the downstream apoptotic cascade, leading to apoptotic cell death [13, 23, 39, 92]. Furthermore, osmotic influx of water through these pores swells mitochondrial matrix and ceases ATP production, disabling ATP-dependent ion exchangers/pumps. The devastated cellular ion homeostasis eventually leads to plasma membrane rupture and necrotic cell death [2, 11].

There are no significant abnormalities in the heart under basal conditions in TRIC-A^*−*/*−*^ mice. However, heart from TRIC-A-deficient mice showed altered SR Ca^2+^ regulation such as (SR Ca^2+^ overload) under stress conditions. For example, chronic treatment with β-adrenergic receptor agonist isoproterenol (ISO) leads to extensive fibrosis development in the TRIC-A^*−*/*−*^ heart. Such effect is likely caused by increased death of cardiomyocytes and fibrotic remodeling, which may be linked to the overload of SR Ca^2+^ associated with the ablation of TRIC-A. SR Ca^2+^ overload due to TRIC-A depletion likely exacerbates mitochondria Ca^2+^ overload, promoting mitochondria ROS production and facilitating apoptosis and/or necrosis of the TRIC-A^−/−^ cardiomyocytes. However, the underlying mechanism is still largely unknown. Therefore, it is worthwhile to further explore the role of TRIC-A in Ca^2+^ mediated SR-mitochondrial crosstalk.

## Localization of TRIC-A in the nuclear envelope suggests potential transcriptional function

TRIC-A is not only located in the ER/SR membrane but also heavily expressed in the membrane of the nuclear envelope of muscle cells [101]. Although the exact function of TRIC-A in the nuclear envelope remains unknown, pioneer studies by Schirmer’s group provided important insights [70]. They screened the nuclear envelope transmembrane proteins (NETs) that mediated the global changes in actively transcribing genomic loci during myoblast differentiation and identified TRIC-A as one of the NETs directing specific chromosomal regions to the nuclear periphery for transcriptional repression during myogenesis [70]. To investigate whether the dysfunction of NETs like TRIC-A contributes to the pathogenesis of hereditary muscular diseases, they acquired primary myoblasts/fibroblasts culture derived from Emery–Dreifuss muscular dystrophy (EDMD) patients. Interestingly, TRIC-A displayed the most notable change of distribution among the 8 candidate NETs in those patients. Most patient samples exhibited moderate redistribution of TRIC-A favoring the SR localization [45]. Recently, they also identified TRIC-A mutations (N260D and N260 deletion) which alter the redistribution of target genes to the nuclear periphery during myogenesis. This result further confirms that abnormalities in TRIC-A-mediated chromosomal repositioning can be an etiological factor of EDMD [59].

Another possible role of TRIC-A on the nuclear envelope could be related to nuclear Ca^2+^ regulation. Since TRIC-A is known to regulate ER/SR Ca^2+^ signaling, its role in the nuclear Ca^2+^ regulation is plausible. It has been shown that Ca^2+^ signaling in the nucleoplasm regulates events that are distinct from the ones mediated by cytosolic Ca^2+^ [25]. For example, cardiomyocyte nucleus contains its own Ca^2+^ store called nucleoplasmic reticulum that expresses RyR and IP_3_R [25, 54]. IP_3_-induced Ca^2+^ release from nucleoplasmic reticulum facilitates protein kinase C (PKC) translocation to the nuclear envelope [25]. Abnormal nuclear Ca^2+^ signaling is also involved in cardiomyocyte hypertrophy. Diminishing Ca^2+^ level in the nuclei leads to swelling of the nuclei in neonatal cardiomyocytes, accompanied by increased calcineurin expression and increased nuclear enrichment of NFAT [34]. It is well known that calcineurin/NFAT signaling cascade plays a critical role in activating the transcription of genes involved in cardiac hypertrophy, such as β-myosin heavy chain (β-MHC) [34, 62]. Recently, we confirmed the noticeable expression of TRIC-A on the periphery and the membrane invaginations of the nuclear envelope of cardiomyocytes (unpublished data). Unlike the skeletal muscle cells, which form a smooth oval shape of the nuclear envelope, cardiomyocytes develop apparent membrane invaginations on the nuclear envelope. Researchers have speculated that such invagination could play a role in gene regulation [104], although the detailed mechanism is still lacking. TRIC-A locates directly on the invagination membrane structure of the nuclear envelope, suggesting it may play a role in nuclear Ca^2+^ regulation and gene translational regulation. Further studies are required for understanding the exact role that TRIC-A plays in the nuclear envelope of cardiomyocytes. The potential of TRIC-A regulating nuclear Ca^2+^ signaling would be an interesting direction for future investigation.

Table 1 summarizes our current understanding for the role of TRIC-A in cardiac, smooth and skeletal muscles, and the phenotypes associated with knockout of TRIC-A or genetic mutation in TRIC-A. In addition to supporting the counter current movement associated with intracellular Ca release, TRIC-A can also interact with RyR channels to directly or indirectly modulate ER/SR Ca homeostasis and crosstalk with mitochondria.

**Table 1:** Phenotypes and mechanism in different tissues

Tissue	Mechanism	Phenotype	Publication
Cardiac muscle	a) Counter-current b) Regulation of RyR_2_ c) Regulation of STIM1 mediated SOCE.	Stress-induced cardiac arrhythmia Stress-induced cardiac fibrosis	Zhou et al., 2020 Shrestha et al., 2020
Smooth muscle	a) Counter-current b) Regulation of RyR-IP_3_R	Hypertension	Yamazaki et al., 2011
Skeletal muscle	a) Counter-current b) Regulation of RyR_1_ c) Nuclear action for gene transcription	Irregular muscular contractile force Emery–Dreifuss muscular dystrophy	Zhao et al., 2010 El-Ajouz et al., 2017 Robson et al., 2016

## Conclusion

**Fig. 1:**
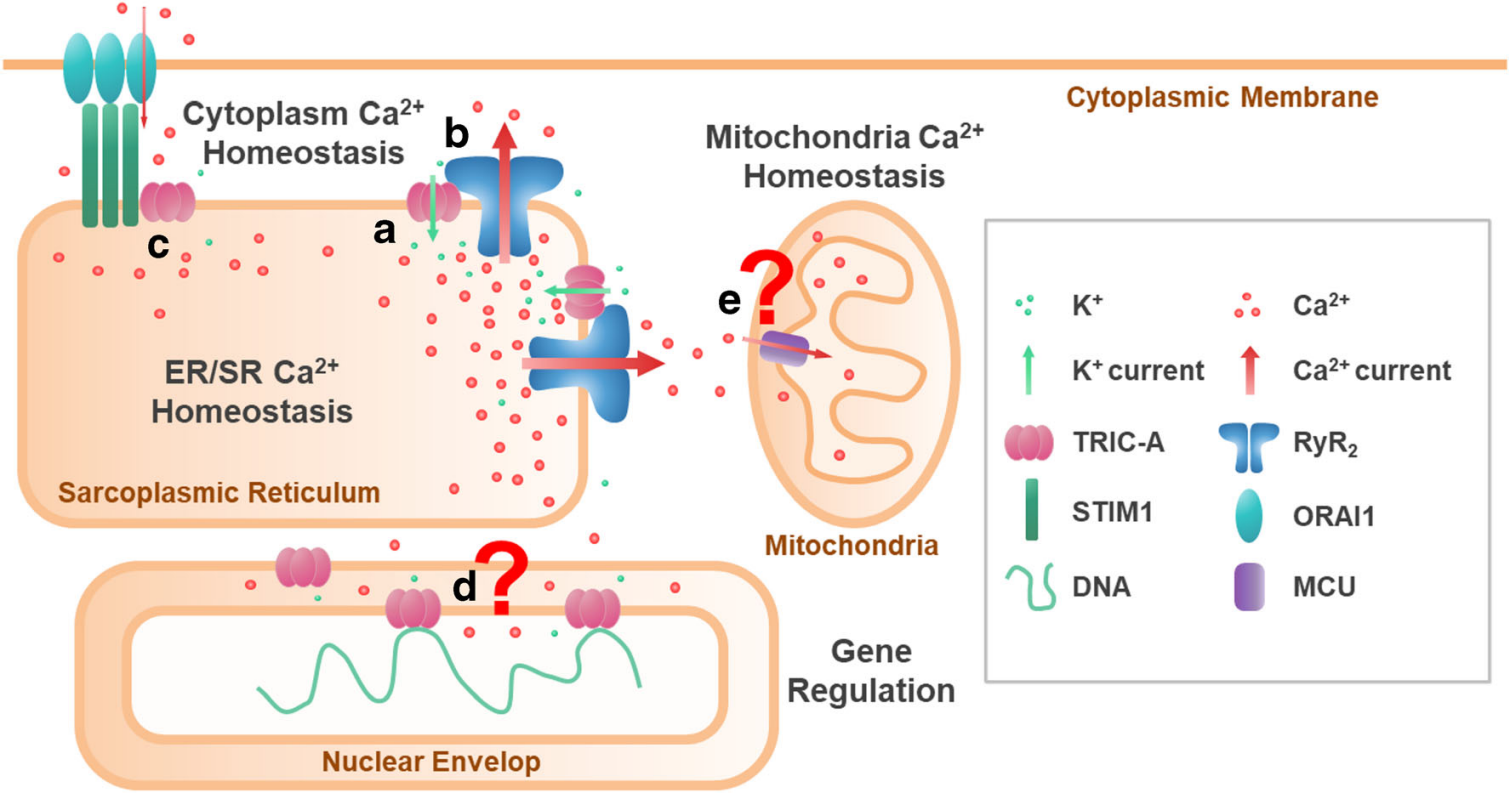
Multi-functional role of TRIC-A in regulating Ca^2+^ signaling in cardiomyocytes. (a) TRIC-A channels are predominantly localized to the ER/SR, providing counter-current K^+^ movement (green arrows) for SR charge compensation during RyR_2_-mediated Ca^2+^ release (thick red arrows). (b) TRIC-A channels directly interact with RyR_2_ through its carboxyl terminal tail to modulate RyR_2_ opening status. (c) Association between TRIC-A and STIM1 has been proposed to control store-operated Ca^2+^ entry. (d) TRIC-A is found in the nuclear envelope, while its role in modulation of nuclear Ca^2+^ signaling and gene transcription remains largely unknown. (e) TRIC-A may function to modulate Ca^2+^ signaling crosstalk from SR to mitochondria to match the metabolic demand of myocardial workload under physiologic or pathologic conditions

We depict a diagram to demonstrate the proposed functions of TRIC-A in cardiomyocytes (Fig. 1). Although there are still debates about whether TRIC channels carry the essential counter-current during RyR channel–mediated Ca^2+^ release, the identification of direct interactions between TRIC-A and RyR_2_ support a pivotal role of TRIC channels in regulating ER/SR Ca^2+^ homeostasis. Due to the growing interest in the physio-pathological roles of TRIC channels, novel regulatory mechanisms mediated by TRIC-A have started to emerge, including direct control of RyR_2_ activity, SOCE, SOICR functions, and its potential role in modulating mitochondria Ca^2+^ signaling. In addition to SR/ER localization, TRIC-A is also identified on the nuclear envelope and may participate in excitation-transcriptional regulation of genes involved in myogenesis and adaptive responses of the muscle and heart under physiologic and pathophysiologic settings.
